# A plant from the altiplano of Northern Chile *Senecio nutans*, inhibits the *Vibrio cholerae* pathogen

**DOI:** 10.1186/s40064-016-3469-6

**Published:** 2016-10-13

**Authors:** Adrian Paredes, Yanett Leyton, Carlos Riquelme, Glauco Morales

**Affiliations:** 1 Laboratorio Mesocosmos Marino, Centro de Bioinnovación de Antofagasta (CBIA), Departamento de Biotecnología, Facultad de Ciencias del Mar y Recursos Biológicos, Universidad de Antofagasta, Av. Universidad de Antofagasta, 02800 Antofagasta, Chile; 2 Laboratorio Química Biológica, Instituto Antofagasta (IA), Universidad de Antofagasta, Av. Universidad de Antofagasta, 02800 Antofagasta, Chile

**Keywords:** Chachacoma, Antimicrobial activities, *Vibrio Cholerae*, *Senecio nutans*, Essential oils

## Abstract

**Background:**

In the altiplano of Northern Chile the plant *Senecio nutans* is habitually used as an infusion to relieve the effects of altitude sickness (locally known as “puna”). It is also used to alleviate the bronchitis, whooping cough, asthma, stomachache, tiredness and fever. The extreme conditions under which these plant grow and scientific data that shows the inhibiting potential of the essential oils of plants of the genus *Senecio* represents great potential in the study of their application to control pathogens like *Vibrio Cholera*.

**Methods:**

The essential oil from aerial parts of *S. nutans* was isolated by hydrodistillation and the chemical composition characterized by GC–MS analyses. The antibacterial potential and determination of MIC value, was estimated in both micro and macro dilution method.

**Results:**

The GC–MS analysis of essential oil of *S. nutans* showed the presence of methyl cinnamate (44.9 %), *p*-cymenol (27.2 %), and terpinen-4-ol (6.8 %), α-terpineol (4.1 %), *t*-cadinol (3.5 %), methyl hydrocinnamate (2.1 %), δ-cadinene (2.0 %), *p*-cymene (1.9 %), γ-terpinene (1.8 %), α-cadinol (1.6 %), *cis*-sabinene hydrate (1.1 %), caryophyllene (0.9 %), β-pinene (0.8 %), and α-terpinene (0.6 %) as major components. Moreover, the oil of *S. nutans* exhibited an important antibacterial activity with a diameter of inhibition zone growth of 22 mm and the MIC value of 0.4 mg/mL against pathogenic bacteria *V. cholerae*.

**Conclusions:**

The results show for the first time the antibacterial activity of the essential oils of *S. nutans* against the *V. cholerae* pathogen, an activity that can be applied as a preventive treatment against the action of pathogen.

## Background

The increasing resistance developed by microorganisms to treatment with antibiotics has affected both humans and the agricultural and aquacultural industry (Broszat and Grohmann 2014). The latter has intensified the search for new antimicrobials to control pathogenic agents. A natural source of antimicrobials agents is provided by plants (Fredes and Montenegro 2013). Essential (volatile) oils of herbs and their components, products from the secondary metabolism of a plant, are complex mixtures of various compounds, usually monoterpenes, sesquiterpenes, phenylpropanes, low molecular weight aliphatic compounds, and others (Benites et al. 2011; Kahriman et al. 2011; Oladipupo and Adebola 2009). The biological activity of essential oils is centered on antimicrobial, anticancer, analgesic, antioxidant, anti-inflammatory, and antithrombotic activities, (Adorjan and Buchbauer 2010; Bakkali et al. 2008). These properties make essential oils the raw materials in various fields such as cosmetics (perfumes and aromatherapy), food (spices), and pharmaceutical (phytotherapy) industries and have many applications in folk medicine (Adorjan and Buchbauer 2010; Citarasu 2010).

*Senecio* is one of the most widely spread genera in the world, with about 3000 species. In South America, Chile and Argentina have the largest numbers, with approximately 300 species each (Bolzan et al. 2007; Moreira-Muñoz and Muñoz-Schick 2007). From the chemical standpoint this genus is characterized by producing pyrrolizidine alkaloids, and its species are the main producers of these compounds with well-known hepatotoxic and carcinogenic activity, but not all *Senecio* species produce these kinds of compounds (Bolzan et al. 2007; Echeverría and Niemeyer 2012). Other constituents of interest from *Senecio* are diterpenes, sesquiterpenes with eremophilane, cacalol, bisabolane, silphinene, caryophillane, humulane, germacrane, triterpenes, *p*-hydroxyacetophenones derivatives, benzofurane and flavonoids (Morales et al. 1986, 1996, 2000; Portero et al. 2013; Ruiz-Vasquez et al. 2015; Wang et al. 2013), and they are also characterized by the presence of polyfructanes and sesquiterpene lactones that are attributed antimicrobial activity (Albayrak et al. 2014; Yanga et al. 2011).

The leaves of *Senecio nutans* are used in the Andes of Northern Chile as an infusion to lower blood pressure counteract the effects of altitude sickness, locally known as “puna.” Other uses of this species are to alleviate the discomfort caused by cold, bronchitis, whooping cough, asthma, stomachache, tiredness, and fever. Sometimes it is also used as a condiment (Carod-Artal and Vázquez-Cabrera 2007; Giberti 1983; Marcía et al. 2005; Villagrán and Castro 2003; Villagrán et al. 2003; Zardini 1984). Species of the genus *Senecio* possess the largest medicinal value for these Andes communities, and studies on the biological activities attributed to them are scarce.

The bacteria *Vibrio cholerae* can be found free or forming biofilms, in association with corals, fish, molluscs, algae, shrimp, and zooplankton, and even in the intestinal contents of these organisms (Leyton and Riquelme 2008). The pathogen *V. cholerae* is the agent that causes the gastrointestinal disease called cholera, which can expand rapidly as an epidemic and/or pandemic through the ingestion of contaminated water and/or food (Mala et al. 2013). It is a Gram-negative and there are around 200 recognized O serotypes, but only serotypes O1 and O139 are associated with serious cases of cholera (Bauman 2012; Hendriksen et al. 2011), manifested by symptoms such as gastroenteritis, skin infections, septicemia, and even death in the case of immunosuppressed patients (Liu et al. 2014; World Health Organization 2014).

In view of the need to control the diseases caused by the pathogenic bacteria *V. cholerae*, and based on the scientific background that shows the inhibiting potential of the essential oil extracts of plants of the genus *Senecio*, the present study had the purpose of evaluating the inhibiting activity of the essential oils of *S. nutans* against the *V. cholerae* pathogen as a future potential application of the components of these extracts to control this pathogen.

## Methods

### Collection of botanical samples

The leaves of *S. nutans* were collected from plants growing in a natural population in nearby the locality of Toconce (22°15′11.16″S, 68°5′44.68″W, at 3788 m.a.s.l.) in April of 2014 at II Región of Antofagasta, Chile. The specimen was authenticated by Dr. Roberto Rodríguez, Departamento de Botánica, Facultad de Ciencias Naturales y Oceanográficas, Universidad de Concepción, Concepción, Chile. A sample was kept for the herbarium collection, under register CONC 139.929.

### Isolation of essential oil (EO)

The essential oils were obtained from 300 g of ground fresh plants by hydrodistillation during 3 h in a Clevenger-type apparatus, and was collected from the aqueous phase using ethyl ether extraction. The oil was sealed in a glass vial and refrigerated at 4 °C to avoid its decomposition by light or heat. The yield of oil was calculated in terms of the obtained oil mass and plant mass used.

### Gas chromatography: mass spectrometry

The determination and quantification of the composition of the essential oil was performed by gas chromatography coupled with mass spectrometry (GC–MS) using an Agilent 5973N GC/MS system, with a fused silica capillary column (30 m × 0.25 mm coated with DB-5, film thickness 0.25 μm), the flow rate was 1 mL/min with helium carrier gas at 7 psi, injector port at a temperature of 250 °C and a split ratio of 1:30 and oven temperature programmed from 60 to 280 °C at 2 °C per min, injection of 2 μL of sample (10 % *n*-hexane).

The mass spectrometer conditions were: electron impact mode (EI) 70 eV, ion source at 230 °C, quadrupole analyzer 150 °C, a scanning range of *m/z* = 35–450. An HP MSD Enhanced ChemStation Software module controlled all parameter. Identification of components were made by comparing retention indices (RI) according to the Wiley Library and NIST 2000 database.

### Getting the pathogenic strain

The pathogenic strain *Vibrio cholerae tor1* was obtained from the strain collection of the Laboratorio Mesocosmos Marino, Centro de Bioinnovación de Antofagasta, Facultad de Ciencias del Mar y Recursos Biológicos, Universidad de Antofagasta. The strain was kept in a cryo-pearl collection and in culture plates with Trypticase soy agar, TSA (Oxoid Ltd, Basingstoke, Hampshire, England) under axenic conditions at 20 ± 1 °C.

### Antibiogram of essential oil from *S. nutans* against *V. cholera*

Antimicrobial activity testing was carried out using a disc-diffusion method. Petri dishes were prepared using Mueller–Hinton agar (38 g/L with addition of 2 % NaCl). A 100 μL aliquot of the pathogenic bacteria was used to inoculate a Trypticase soy culture broth (TSB), incubating at 20 °C during 12 h. Sterile filter paper discs of 6 mm in diameter were impregnated directly with 10 μL of the oil (which correspond to 9.28 mg of essential oils *S. nutans*). A 100 μL sample was removed from the 12-h culture and it was streaked on the surface of the solid medium using a sterile loop. The disc with the product was then placed on the surface seeded with the pathogen. The control antibiotics used were Chloramphenicol (CL) (30 μg/disc), Streptomycin (S) (10 μg/disc), Sulfamethoxazol/Trimetoprim (SXT) (25 μg/disc), and Cefotaxime (CTX) (30 μg/disc). Each treatment was performed in triplicate. The plates were incubated at 37 °C during 24 h. A halo >5 mm around the filter caused by the absence of bacterial growth was considered as inhibition.

### Identification of the inhibiting concentration of *S. nutans*

The minimum inhibitory concentration (MIC) was determined according to the micro-dilution in broth method described by (Rejiniemon et al. 2014; Wiegand et al. 2008), with the following modifications: (a) sterile 96-well plates were used, (12 mm × 8 mm. Costar^©^, 96 Well Cell Culture Cluster, Corning Incorporated); (B) seawater, previously filtered to 0.2 μm and kept at 4 °C, was used as the test medium; (C) the essential oil concentration range studied was 0.05–12.8 mg/mL, obtained from three stock solutions in ethanol: Stock Solution A—50 mg/mL (0.075 g of sample in 1.5 mL of ethanol); Stock Solution B—12.8 mg/mL (89.6 μL of Stock Solution A were diluted in 260 μL of solvent); Stock Solution C—0.8 mg/mL: (31.25 μL of Stock Solution B were diluted in 469 μL of solvent. Then the desired concentrations were placed in each well considering a final volume of 200 μL.

The bacterial inoculum was added at a concentration of 1 × 10^8^ CFU/mL, obtained from an 18-h culture in Trypticase soy broth. Each plate was incubated at 37 °C for 24 h. The controls were seawater (medium in which the bacteria were diluted and inoculated in each well), and ethanol (solvent used to dilute the essential oil), with one control for each sample concentration. The resulting turbidity was observed and MIC value was determined to be where growth was no longer visible by assessment of turbidity by optical density reading at 620 nm with a Tecan^©^ Sunrise 96 well Microplate Readers. All essential oil concentrations and controls used were analyzed in triplicate.

### Bacterial growth in the presence of *S. nutans* essential oil

Aliquots of 10, 30 and 60 μL of *S. nutans* essential oil were added directly to test tubes, getting concentrations of 1, 3 and 6 mg/mL. Bacteria without essential oil (CT), and bacteria plus Chloramphenicol (CT CL) in concentration of 1 mg/mL were used as positive control. From an 18-h culture of the bacteria in TSB, an initial concentration of 1 × 10^3^ CFU/mL was inoculated. The treatments and control were made in triplicate. Bacterial growth inhibition was evaluated at 48 h of culture by counting viable bacteria in TSA plates that were incubated at 37 °C during 48 h and the bacterial count was expressed in colony forming units per milliliter (CFU/mL).

### Statistical analysis

The results were reported as mean ± standard error. One way ANOVA and Tukey *post Hoc* multiple comparison test were used to analyze data with the sofware GraphPad Prism, version 5.0 for Mac OS X. The level of significance was set at *P* < 0.05.

## Results

### Components of *S. nutans* essential oil

The yield of essential oil from *S. nutans* was 0.37 % (w/w based on fresh plants). The oil has a yellow color and a strong smell. The composition obtained by GC–MS (Table 1) showed that the main compounds were: methyl cinnamate (44.9 %), *p*-cymenol (27.2 %), terpinen-4-ol (6.8 %), α-terpineol (4.1 %), *t*-cadinol (3.5 %), methyl hydrocinnamate (2.1 %), δ-cadinene (2.0 %), *p*-cymene (1.9 %), γ-terpinene (1.8 %), α-cadinol (1.6 %), *cis*-sabinene hydrate (1.1 %), caryophyllene (0.9 %), β-pinene (0.8 %), and α-terpinene (0.6 %). These compounds represent approximately 98.7 % of the composition of the essential oil. The remaining 1.3 % of the compounds corresponded to those with lower abundance of 0.5 %, as α-phelandrene (0.4 %), *trans*-piperitol (0.4 %), linalool (0.3 %), α-humulene (0.1 %) and thymol (0.1 %).

**Table 1:** Percentage of chemical composition of the essential oil from the aerials part of *S. nutans*

No	Components^a^	RI^b^	%^c^	Methods of identification^d^
1	β-pinene	980	0.8	MS, RI
2	α-phellandrene	1006	0.4	MS, RI
3	α-terpinene	1018	0.6	MS, RI
4	*p*-cymene	1026	1.9	MS, RI
5	*cis*-sabinene hydrate	1068	1.1	MS, RI
6	γ-terpinene	1070	1.8	MS, RI
7	linalool	1099	0.3	MS, RI
8	terpinen-4-ol	1182	6.8	MS, RI
9	*p*-cymenol	1187	27.2	MS, RI
10	α-terpineol	1194	4.1	MS, RI
11	*trans*-piperitol	1206	0.4	MS, RI
12	methyl hydrocinnamate	1281	2.1	MS, RI
13	thymol	1290	0.1	MS, RI
14	methyl cinnamate	1372	44.9	MS, RI
15	caryophyllene	1410	0.9	MS, RI
16	α-humulene	1463	0.1	MS, RI
17	δ-cadinene	1517	2.0	MS, RI
18	*t*-cadinol	1625	3.5	MS, RI
19	α-cadinol	1670	1.6	MS, RI
	Total		100	
	Monoterpenes hydrocarbons		21.1	
	Oxygenated monoterpenes		26.3	
	Sesquiterpenes hydrocarbons		15.8	
	Oxygenated sesquiterpenes		10.5	
	Aromatic hydrocarbon		5.3	
	Oxygenated aromatic hydrocarbon		21.0	

^a^Compounds presented according to the elution order in the column DB5

^b^Retention Index relative on the DB5 column

^c^Percentage based on FID peak area normalization

^d^MS: compounds were tentatively identified by comparison with mass spectra data (MS) obtained from NIST/EPA/NIH and Wiley library; RI: confirmed by comparison with kovat´s index on DB-5 column

### Antibiograms of *S. nutans* essential oil against *V. cholerae*

**Fig. 1:**
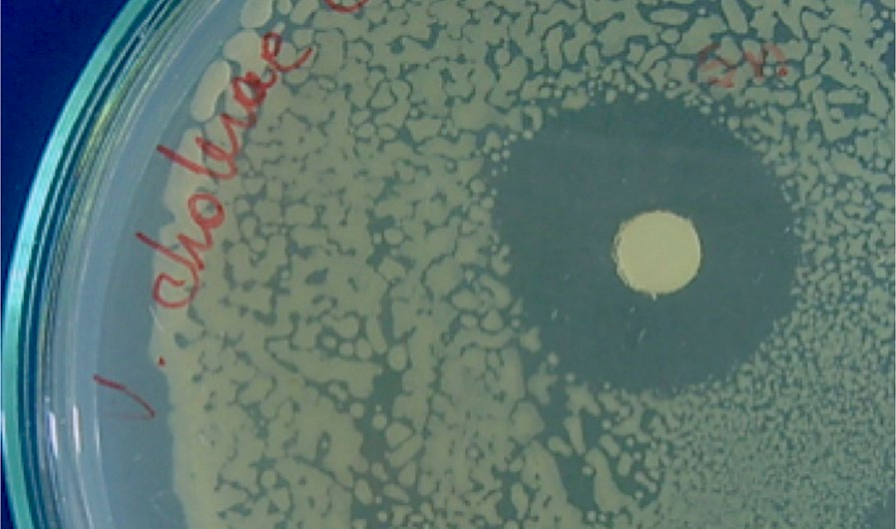
Representative image of antibiograms of the essential oil (10 μL) of *S. nutans* against *V. cholerae* pathogen by disc diffusion method

**Fig. 2:**
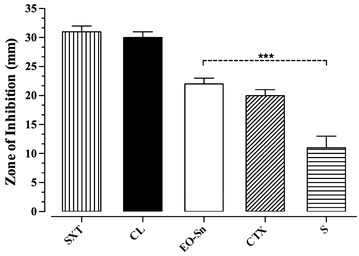
Inhibitions zones (mm) of essential oil of *S. nutans* and antibiotic used as control. Data represent the means and standard error of the means (*n* = 3). The symbol *** shows significant differences (*P* < 0.05) respect to the control antibiotic Streptomycin (S) (10 μg/disc). The others antibiotics used were: Chloramphenicol (CL) (30 μg/disc), Sulfamethoxazol/Trimetoprim (SXT) (25 μg/disc), and Cefotaxime (CTX) (30 μg/disc)

The essential oil of *S. nutans* showed a 22-mm diameter inhibition halo against the pathogenic bacteria *V. cholerae* (tor1) (Fig. 1), while the controls showed Chloramphenicol 30 mm, streptomycin 11 mm, sulfamethoxazol/trimetoprim 31 mm, and cefotaxime 20 mm diameter halos. The inhibition halo of *S. nutans* remained for 7 days, and growth appeared in the inhibition zone after that period. The inhibiting activity shown by the essential oil of *S. nutans* had a similar diameter to that shown by the control antibiotic cefotaxime (*P* > 0.05) which had a 20 mm diameter halo. Additionally, the essential oil showed a significantly greater inhibition (*P* < 0.05) than the control streptomycin, which had an 11 mm diameter halo (Fig. 2).

### Inhibiting concentration of *S. nutans*

**Fig. 3:**
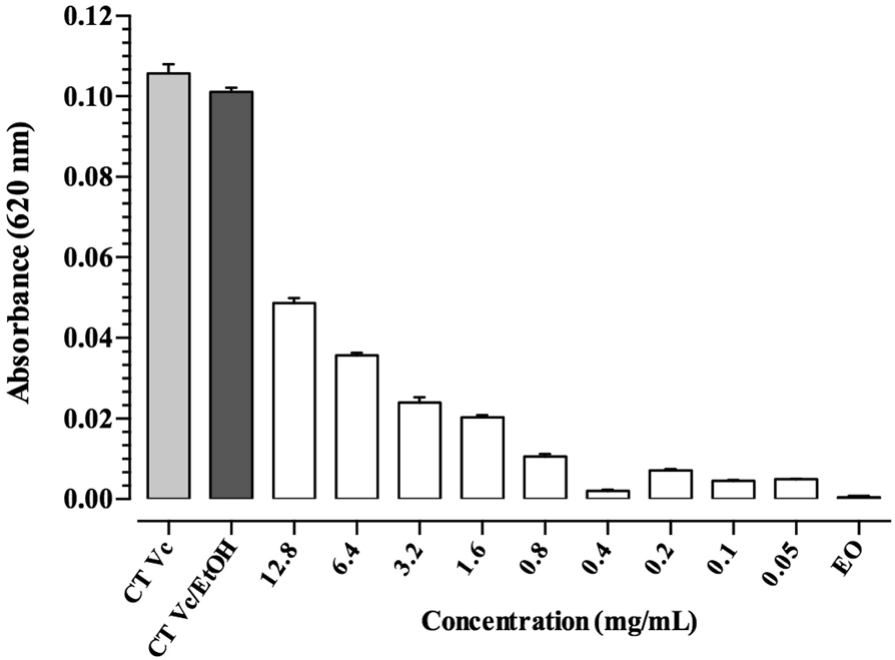
Inhibitory bacterial concentration by essential oil of *S. nutans*. The *bars* represent the means and standard error (*n* = 3). CT (bacteria without essential oil), CT/EtOH (bacteria plus ethanol used to dissolve the essential oil) and EO (the essential oil of *S. nutans* in solution without bacteria as control)

The results in multi-well plates showed that when the bacterial pathogen was exposed to the essential oil of *S. nutans*, its growth was inhibited during the first 24 h of culture in all the treatments, showing significant differences at all concentrations with respect to the control (*P* < 0.05) confirming the antimicrobial property. Additionally, it was observed that the most effective concentrations of the essential oil of *S. nutans* for inhibiting Gram-negative bacteria *V. cholerae*, are in the range of concentrations of 0.05–0.4 mg/mL, showing no significant difference between them (*P* > 0.05). The MIC value for the essential oil of *S. nutans* was 0.4 mg/mL, this value showed no significant differences with respect to control that contained only the essential oil (*P* > 0.05), showing a clear inhibition of the growth of the bacteria (Fig. 3).

### Bacterial growth in the presence of essential oil of *S. nutans*

**Fig. 4:**
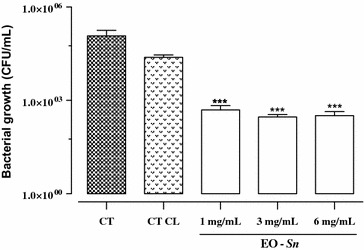
Bacterial growth in the presence of essential oil of *S. nutans*. CT (untreated bacteria); CT CL (bacteria treated with Chloramphenicol (1 mg/mL); 1, 3 and 6 (bacteria treated with 1, 3 and 6 mg/mL of essential oil). The symbol *** shows significant differences (*P* < 0.05) respect to the controls (CT and CT CL). Data represent the means and standard error of the means (*n* = 3)

Bacterial growth in the presence of three essential oil concentrations of 1, 3 and 6 mg/mL was evaluated. The growth was measured in CFU/mL of the pathogenic bacteria in the presence of the essential oil (Fig. 4). The results showed that the three concentrations showed inhibition of the pathogen’s growth with significant differences with respect to the controls (*P* < 0.05). The greatest inhibition was seen with 6 mg/mL, yielding a count of 2.5 × 10^2^ CFU/mL, followed by 3 mg/mL with a count of 3.0 × 10^2^ CFU/mL, and 1 mg/mL with a count of 5.0 × 10^2^ CFU/mL. The statistical analysis showed no significant differences (*P* > 0.05) between the antibacterial activities of these three concentrations of essential oil. The controls yielded the following results: CT 1.2 × 10^5^ CFU/mL and CT CL 2.7 × 10^4^ CFU/mL.

## Discussion

In the present study, the essential oil of *S. nutans* show a similar composition and a higher yield than described by Belaunde et al. (2007), De Feo et al. (2003). The differences in the composition of the essential oils of *S. nutans*, can be attributed to the chemotype variation, influenced by the geographic location, seasonal and climatic conditions and high levels of ultraviolet radiation in the desert regions where it inhabits this species (Reyes-Jurado et al. 2014). Analyses GC–MS identified a total of 19 components with one of the most abundant being methyl cinnamate (44.9 %) followed by *p*-cymenol (27.2 %). These two components together represent approximately the 72 % of the total composition.

The essential oil of *S. atacamensis,* another species of *Senecio* that grows in the north of Chile, has been attributed antimicrobial properties against human pathogens by disc diffusion and dilution tests. The most abundant monoterpenes were α-terpinene, α-phellandrene, and *p*-cymene (Benites et al. 2011), which differ from the main components of *S. nutans.*

The inhibitory activity of the essential oil can be attributed to the monoterpene hydrocarbons that have been reported as the main components of the essential oils of various species of the genus *Senecio* (Kahriman et al. 2011). Some authors state that oils containing high percentages of monoterpene compounds (α-terpinene, α-phellandrene, *p*-cymene, sabinene) have significant antimicrobial properties (Benites et al. 2011). On the other hand, the antimicrobial activity of the essential oils is attributed to the presence of terpenoids, followed by those that contain alcohol groups, those that have aldehyde groups, and finally those that contain ketone groups (Yanga et al. 2011). The combination of these compounds present in the essential oils can act collectively, causing antibacterial activity by synergistic effect. In other cases, an antagonistic effect had been observed in the components of essential oils, significantly reducing it`s activity (Bassolé and Juliani 2012). The mechanism of the antibacterial action of the essential oils, mainly that of the terpenoids, is associated with the bacterial cell membrane, whose permeability to small ions increases, affecting its structural stability, destabilizing the packing of the lipidic bilayer, and causing the death of the bacterial cell. For example, it been described that terpinen-4-ol and α-terpineol induced K^+^ leakage from bacterial cells in different concentration, being more active at lower concentration terpinen-4-ol. The mode of antimicrobial action of this compound is attributed to the general characteristic of oxygenated terpenes and their ability to cause membrane permeability, K^+^ leakage and reduction of membrane potential, collapsing of the proton pump, and depletion of the ATP pool (Reyes-Jurado et al. 2014). The compounds α-terpineol and terpinen-4-ol, which are present in different proportions in the essential oil of *S. nutans*, could explain in part the mechanism of action on pathogenic bacteria of *V. cholerae.*

## Conclusions

The results show for the first time the antibacterial activity of the essential oil of *S. nutans* against the *V. cholerae* pathogen, which represents a great applicability to preventive treatments of pathogens in aquacultural systems. This research opens the way for many new studies, such as those aimed in gaining essential oils of *S. nutans* in greater volumes, confirming the activity of the product in cultures of contaminated organisms, and evaluating whether the antibacterial activity has a direct relation with a specific predominant compound or a synergy of products that compose the essential oil. Compared to artificial chemicals or synthetic additives, essential oils have been recognized as effective decontaminants against various pathogens without adverse effect to human health (Tongnuanchan and Benjakul 2014; Wang et al. 2015). Additionally, the drugs of natural origin represent an interesting approach to limiting the appearance and propagation of hard to treat microorganisms. Moreover, the use of oils from plants from the altiplano that grow under extreme conditions is an interesting field and scarcely studied. These studies may have direct application in aquaculture, agriculture and phytotherapy, which has a high impulse at national level. The use of essential oils has certain advantages associated with a: reduced genotoxicity (even after prolonged used), the ability to act on multiple cellular targets, low cost for the productions and the most important, a less toxicity.
